# Genetic and Functional Characterization of Novel Brown-Like Adipocytes Around the Lamprey Brain

**DOI:** 10.3389/fcell.2021.674939

**Published:** 2021-07-01

**Authors:** XiaoLuan Xu, AnQi Ma, TieSong Li, WenXue Cui, XueFeng Wang, Jun Li, Qingwei Li, Yue Pang

**Affiliations:** ^1^College of Life Sciences, Liaoning Normal University, Dalian, China; ^2^Lamprey Research Center, Liaoning Normal University, Dalian, China

**Keywords:** brown adipocytes, lamprey, immune response, uncoupling protein 2 (UCP2), evolution

## Abstract

During the process of vertebrate evolution, many thermogenic organs and mechanisms have appeared. Mammalian brown adipose tissue (BAT) generates heat through the uncoupling oxidative phosphorylation of mitochondria, acts as a natural defense against hypothermia and inhibits the development of obesity. Although the existence, cellular origin and molecular identity of BAT in humans have been well studied, the genetic and functional characteristics of BAT from lampreys remain unknown. Here, we identified and characterized a novel, naturally existing brown-like adipocytes at the lamprey brain periphery. Similar to human BAT, the lamprey brain periphery contains brown-like adipocytes that maintain the same morphology as human brown adipocytes, containing multilocular lipid droplets and high mitochondrion numbers. Furthermore, we found that brown-like adipocytes in the periphery of lamprey brains responded to thermogenic reagent treatment and cold exposure and that lamprey UCP2 promoted precursor adipocyte differentiation. Molecular mapping by RNA-sequencing showed that inflammation in brown-like adipocytes treated with LPS and 25HC was enhanced compared to controls. The results of this study provide new evidence for human BAT research and demonstrate the multilocular adipose cell functions of lampreys, including: (1) providing material energy and protecting structure, (2) generating additional heat and contributing to adaptation to low-temperature environments, and (3) resisting external pathogens.

## Introduction

There are large number of obesity-related diseases, including type 2 diabetes, insulin resistance, heart disease, dyslipidemia, hypertension and various cancers (Bornfeldt and Tabas, 2011; Harms and Seale, 2013). For many decades, brown adipose tissue (BAT) in mammals, as an important heat-generating tissue, has been considered an attractive target for treating metabolic disease and promoting weight loss (Vitali et al., 2012; Guénantin et al., 2017; Chan et al., 2019).

Brown adipocytes in BAT contain multilocular lipid droplets and are densely packed with mitochondria, promoting energy expenditure. BAT forms prior to other types of fat depots during embryonic development. In human infants, BAT depots have also been found in interscapular regions, but BAT depots in adults regress or are absent (Heaton, 1972; Lidell et al., 2013). Previous studies have demonstrated that most brown adipocytes originate from precursor cells in embryonic mesodermal somites that can also develop into skeletal muscle cells (Seale et al., 2008; Lepper and Fan, 2010; Sanchez-Gurmaches et al., 2012). In rodents, the major BAT depots are located in the interscapular, axillary and cervical pad regions (Wu et al., 2013; Kajimura and Saito, 2014). In sea lampreys, males exhibit the secondary sexual characteristic of thermogenic adipose tissue, which immediately increases heat production during mating (Chung-Davidson et al., 2013). In progenitor cell populations, myogenic factor 5 (Myf5) and Pax7 expression is considered a selective marker of skeletal myoblast (Lepper and Fan, 2010; Sanchez-Gurmaches et al., 2012), and brown adipocyte precursor cells express a gene profile similar to that of muscle (Timmons et al., 2007); in addition, they possess related mitochondrial proteomes (Forner et al., 2009). Brown adipocytes in BAT are defined as cells containing multilocular lipid droplet, high mitochondrial content and the expression of brown adipocyte markers, including uncoupling protein-1 (Ucp1) (Kopecky et al., 1995; Bettini et al., 2019), cell death-inducing DNA fragmentation factor alpha-like effector A (Cidea) (Zhou et al., 2003; Toh et al., 2008), *type II iodothyronine* 5′-*deiodinase* (Dio2) (Chan et al., 2019), cytochrome c oxidase polypeptide 7A1 (Cox7a1) and cytochrome c oxidase subunit VIIIb (Cox8b) (Chan et al., 2019) as well as transcriptional regulators, including PR domain zinc finger protein 16 (PRDM16) (Forner et al., 2009), peroxisome proliferator-activated receptor-γ coactivator 1-α (Pgc-1α) (Hondares et al., 2006; Hallberg et al., 2008), and peroxisome proliferator-activated CCAAT/enlmnccr-binding protein B (C/EBPb) (Tanaka et al., 1997; Carmona et al., 2005; Karamanlidis et al., 2007) and receptor alpha (PPARα) (Tai et al., 1996; Siersbæk et al., 2012).

Lampreys have recently received much attention in the fields of comparative development and genomics. Lampreys belong to the jawless vertebrates and are one of the two most ancient groups of vertebrate representatives. The transition from jawless to jawed vertebrates was caused by some significant evolutionary events, including gene origination and evolution, gene duplication, and cell population and tissue structure changes. Due to its unique evolutionary status, lampreys are essential for exploring the evolution of vertebrates and the origin of different functional mechanisms (Smith et al., 2018; Guan et al., 2019). In addition, lampreys are typical migratory organisms that live in the ocean for part of the year. In autumn, they swim from the ocean into rivers and inhabit the downstream portions of rivers until May or June of the next year. The lampreys travel further upstream in the rivers and lay eggs when the water temperature rises to approximately 15°C. Thus, we wondered how lampreys maintain an elevated body temperature while their body temperature appears to fluctuate with the temperature of the surrounding water. In mammalian BAT, fatty acids are burned to produce heat through the uncoupling of mitochondrial oxidative phosphorylation (Cannon and Nedergaard, 2004). Birds can regulate body temperature via the non-shivering thermogenesis of muscles (Mozo et al., 2005). Fish can maintain higher brain temperatures than the ambient temperature through the ATP-dependent Ca^2+^ cycle in the sarcoplasmic reticulum of heating cells around the brain (Block, 1987). Moreover, many animals use stored fat as an energy source; for example, *C. elegans* store lipid in their intestinal epithelial cells and *Drosophila* have fat bodies (Imrie and Sadler, 2010). Therefore, the identification of possible thermogenic adipocytes in lampreys was interesting. As the brain tissue of lampreys seems to play a positive role in the spawning act, we examined the morphology and development of the brain tissue and identified the characteristics of these heat-producing fat cells via histology, biochemistry, molecular biology, and transcriptome analyses. To our surprise, the morphology of the brain tissue included multivacuolar fat; this finding led us to speculate that the brain tissue shows differential thermogenic ability.

## Materials and Methods

### Collection and Maintenance of Animals

Adult lamprey specimens of *Lethenteron reissneri*, including both males and females, were captured from the Tong jiang River of China. They were kept in a glass container at a temperature of 4°C. Lamprey handling and all of the experimental procedures were approved by the Animal Welfare and Research Ethics Committee of the Institute of Dalian Medical University (Permit Number: SCXK2008-0002).

### Histology

Lampreys were anesthetized with 0.05% tricaine methanesulfonate (MS-222; 3-aminobenzoic acid ethyl ester, Sigma), and their brain tissues were then stripped and fixed in 10% buffered formalin and embedded in paraffin wax. The paraffin-embedded tissues were sectioned to a thickness of approximately 4 μm. Tissues were then stained with hematoxylin and eosin (HE).

### Isolation of Brown Adipocytes From the Outside Surface of the Adult Lamprey Brain

Brown adipocytes were isolated from the outside surface of the adult lamprey brain and then digested with 0.1% collagenase for 10 min. The cell suspension was passed through a 70 μM cell strainer and centrifuged at 1500 rpm for 5 min to collect the adipocytes. Since lampreys live in cold water, the cells were then cultured at 18°C and expanded in M199 medium supplemented with 10% FBS and 1% penicillin/streptomycin. Cell samples were collected for staining, RNA extraction or immunogenic stimulation.

### Oil Red O Staining

Brown-like adipocytes from the lamprey brain periphery and white adipocytes from the peripheral membrane tissue of the crucian carp brain were stained with 0.3% (w/v) Oil Red O (Sigma–Aldrich, St. Louis, MO, United States) for 6 min at room temperature after being fixed with anhydrous ethanol. Cells that had been counterstained with hematoxylin for 5 min were studied using light microscopy (LSM 780, ZEISS, Germany).

### BODIPY® Lipid Probe Staining

The neutral lipids of brown-like adipocytes were stained with Hoechst (Sigma) for 5 min to visualize cell nuclei and stained with 1:250 BODIPY® Lipid Probes (Thermo Fisher Scientific, United States) for 40 min. Then, the cells were washed three times with PBS and analyzed on a Zeiss Axio Scope A1upright microscope (Carl Zeiss, Inc.).

### Transmission Electron Microscopy

After the tissue was isolated, it was quickly placed into pentanediol fixed solution at 4°C to maintain the tissue cells in the original living state to the greatest extent possible. The tissue was then fixed with pentanediol for 2 h, washed with PBS several times, progressively dehydrated with gradient ethanol and acetone, and embedded in Epon 812. The sections were stained with 2% uranyl acetate (w/v) in 70% methanol (v/v) and 0.5% lead citrate. Observations and image recording of the cells were performed with a JEM-2000EX TEM.

### Quantitative Real-Time PCR (Q-PCR)

Brown-like adipocytes were cultured in M199 medium for 6 h and incubated with LPS (100 μg/mL), 25-hydroxycholesterol (50 μM), adrenaline (1 μM), isoprenaline (10 μM) and norepinephrine (0.5 μM) for 24 h at 18°C. In addition, G418 was used to screen 3T3-L1 cells stably overexpressing UCP2 and control cells, and uninduced precursor adipocytes and differentiated mature adipocytes were collected. Total RNA was extracted with a MiniBEST Universal RNA Extraction kit (TAKARA, China) according to the manufacturer’s instructions, and the RNA was treated with DNase I (TaKaRa, China). Reverse transcription, Q-PCR were performed as previously described (Pei et al., 2016). The sequences of the primers and the accession numbers of the genes selected as brown-like adipocyte markers, regulators and immune molecules are provided in Supplementary Tables 1–3. Each reaction was performed in triplicate, and the data were normalized to those of *L*-*gapdh* as an internal control. Relative gene expression levels in brown-like adipocytes were calculated using the delta Ct (ΔΔCt) method.

### Oxygen Consumption (OCR) and Extracellular Acidification (ECAR) Measurements

Cells were loaded into XF24 islet capture microplates (Seahorse Bioscience) before the experiment, and the optimal cell density (50000 cells/well) was determined experimentally to ensure a proportional response to the probe according to the cell number. The cells were divided into two subsets: one subset was cultured for 8 h at 4°C for cold exposure, and the other subset was cultured for 8 h at 20°C. To determine the absolute respiratory rate, cells were plated in non-buffered DMEM containing 25 mM glucose and 1 mM pyruvate. Measurements were obtained with an Extracellular Flux Analyzer (Seahorse Bioscience, Billerica, MA, United States) to record OCR to represent mitochondrial respiration and ECAR to represent glycolysis.

### Phylogenetic Analyses of the UCP Family

All of the amino acid sequences of *Lethenteron reissneri* UCP family members were obtained from a three-generation sequencing library, and the corresponding sequences of other species were obtained from the Ensemble database and the NCBI database. SMART and Pfam were used to predict the functional domains of UCP family genes. MEME online software was used to analyze the conserved amino acid sequence of the UCP protein family, and 26 different motifs were identified from the results. All collected amino acid sequences of the UCP protein family from different species were input into Clustal X software, and a phylogenetic tree was then constructed in MEGA 7.0 software via the NJ method. A synteny analysis of the UCP protein family was conducted by using Genomicus online software, which can be used not only to establish genetic homology but also to provide clues about the origin of genetic mechanisms.

### Preparation of the LrUCP2 Protein and Anti-L-UCP2 Antibodies

The open reading frame (ORF) of LrUCP2 was subcloned into the pCold I vector. The LrUCP2 protein was purified by a Ni-NTA His-Bind column. Four mice were used for antibody preparation. Each mouse was first injected with 100 μg of protein mixed with complete Freund’s adjuvant (CFA) (Sigma, United States) and subsequently injected with 50 μg of protein mixed with incomplete Freund’s adjuvant. After four injections at 1 week intervals, blood was drawn from the mouse, and antibody purification was conducted using Protein A (GenScript, United States). The ELISA method was used to determine the antibody titers. Briefly, the rL-UCP2 protein was diluted with a coating buffer (1:500) at 4°C overnight, and the mouse antiserum was serially diluted; normal mouse serum (1:50000) was used as the negative control, and PBS was used as the blank control. The ELISA substrate (100 μL/well) was used for color detection, and the OD at 450 nm was detected. The final titer of the obtained antibody was determined.

### Immunohistochemical Staining

Paraffin-embedded brain tissue sections were deparaffinized in xylene and hydrated with ethanol. The tissue sections were incubated with an anti-L-UCP2 polyclonal Ab at a 1/1600 dilution (0.3 μg/mL) for 3 h at room temperature. Normal mouse IgG was used as a negative control. The sections were stained with diaminobenzidine (DAB) and counterstained with hematoxylin. After dehydration, the sections were successively passed through xylene at different concentrations for 15 min and then mounted in neutral resin.

### Cell Culture

The 3T3-L1 cell line was purchased from the American Type Culture Collection and cultured in DMEM with 10% FBS. For adipocytes differentiation, 3T3-L1 cells were grown to confluence, and were treated with medium containing 10% FBS, 0.5 mM isobutylmethylxanthine, 125 nM indomethacin, 1 mM dexamethasone, 20 nM insulin, and 1 nM T3. After 48 h, the cell culture medium was replaced by DMEM containing only 10% FBS, 1 nM T3, and 20 nM insulin. After 2 days, the medium was changed, and the cells were cultured with DMEM until harvest.

### Confocal Microscopy for the Localization of LrUCP2

Firstly, 3T3-L1 cells were cultured overnight in DMEM containing 10% FBS. Then, the cells were transfected with pEGFP-N1 and pEGFP-N1-*ucp2* by using Lipofectamine 3,000 (Life Technologies, Thermo Fisher Scientific, Waltham, MA, United States) in 8-well chamber slides. At 30 h after transfection, confocal microscopy was used to detect the localization of lamprey UCP2 in 3T3-L1 cells.

### Determination of ATP and Free Fatty Acids (FFAs)

The ATP and FFA concentrations of 3T3-L1 cells that overexpressed LrUCP2 with or without induction were determined by using an Enhanced ATP Assay Kit (Beyotime, Shanghai, China) and a Free fatty acid Detection Kit (Solarbio, Beijing, China), respectively.

### RNA-Seq Data Analysis

Brown-like adipocytes were cultured in M199 medium for 6 h, then incubated with LPS (100 μg/mL) and 25-hydroxycholesterol (50 μM) for 12 h and 24 h. 25HC can induce the production of IL-8 and a variety of cytokines and cause a proinflammatory response (Sottero et al., 2007; Gold et al., 2012). It was used to simulate a proinflammatory environment to test whether lamprey brown-like adipocytes have an effect on this. RNA-seq libraries were generated using the NEBNext® Ultra^TM^ RNA Library Prep Kit for Illumina® (NEB, United States) following the manufacturer’s recommendations, and library quality was assessed on the Agilent Bioanalyzer 2100 system. Clustering of the index-coded samples was performed on a cBot Cluster Generation System using a TruSeq PE Cluster Kit v3-cBot-HS (Illumina) according to the manufacturer’s instructions. After cluster generation, the library preparations were sequenced on the Illumina HiSeq platform, and paired-end reads were generated. Raw data were first processed with in-house Perl scripts. In this step, clean data were obtained by removing reads containing adapters or poly-*N* sequences and low-quality reads from the raw data. All downstream analyses were based on clean data with high quality. Transcriptome assembly was accomplished using Trinity (Grabherr et al., 2011) with min_kmer_cov set to 2 by default and all other parameters set to the defaults. The accession number for the RNA-seq sequencing data is PRJNA733594.

### Transcriptome Analyses

Prior to differential gene expression analysis, for each sequenced library, the read counts were adjusted by the edgeR program package through one scaling normalized factor. Differential expression analysis of two samples was performed using the DEGseq (2010) R package. *P*-values were adjusted using *q*-values (Storey and Tibshirani, 2003). A *q*-value < 0.005 and a | log2 (foldchange)| > 1 were set as the thresholds for significantly differential expression. Gene Ontology (GO) enrichment analysis of the differentially expressed genes (DEGs) was implemented by using the GOseq R package-based Wallenius non-central hypergeometric distribution (Young et al., 2010), which can adjust for gene length bias in DEGs. KEGG (Kanehisa et al., 2008) is a database resource for understanding the high-level functions and utilities of biological systems, such as cells, organisms and ecosystems, from molecular-level information, especially large-scale molecular datasets generated by genome sequencing and other high-throughput experimental technologies^1^. We used KOBAS (Mao et al., 2005) software to test the statistical enrichment of differentially expressed genes in KEGG pathways.

### Statistical Analysis

GraphPad Prism 8 (GraphPad Software, La Jolla, CA, United States) was used for all statistical analyses. Differences between treatment groups were determined with Student’s *t*-test. *P* < 0.05 was set as the threshold for significance (^∗^*P* < 0.05, ^∗∗^*P* < 0.01). Each sample was analyzed in triplicate, and the experiment was performed three times. Bar charts were generated to show the means ± SDs of three independent experiments.

## Results

### The Origin and Morphological Characteristics of the Cells Around Lamprey Brain Tissue

**FIGURE 1:**
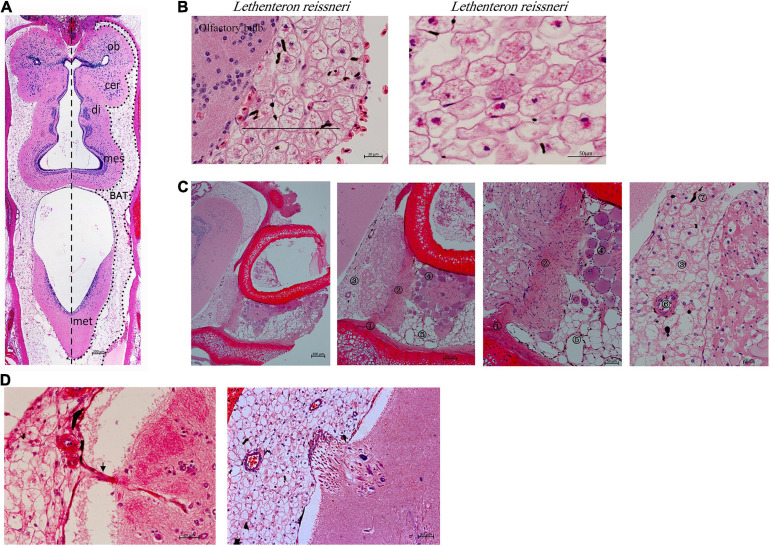
The structure of the lamprey brain and multilocular adipocytes (*Lethenteron reissneri*). **(A)** Coronal section view of the adult lamprey brain. Scale bars, 200 μm. **(B)** The morphology of multilocular adipocytes and the location in the olfactory bulb. di, diencephalon; mes, mesencephalon; met, metencephalon; ob, olfactory bulb; cer, cerebrum. Scale bars, 20 μm or 50 μm. **(C)** Morphological observation of the origin and differentiation of multilocular adipocytes. ①, myofibroblasts; ②, myofibroblasts; ③, multilocular adipocytes; ④, myotube; ⑤, white adipocytes; ⑥, blood vessel; ⑦, pigment cells. The images on the right are partial magnifications of the image on the left. **(D)** The black arrow shows that multilocular adipocytes can extend into brain tissue (left pane). The interaction of multilocular adipocytes with the brain (right pane).

Lampreys have a head and a brain, which is divided into five parts (telencephalon, diencephalon, mesencephalon, cerebellum and rhombencephalon), and the overall shape of the brain is elongated (Figure 1A). Lamprey brain tissues are surrounded by a group of large cells (black line) distributed from the olfactory bulb to the cerebellum, especially in the diencephalon, mesencephalon, and cerebellum (Figures 1A,B). Hematoxylin and eosin staining (Figure 1C) revealed the existence of different types of cells around brain tissues, such as myofibroblasts and the large cells shown in Figure 1B. Spindle-shaped myofibroblasts are attached to the adjacent cartilage, and the myofibroblasts connecting the two sides of the cartilage are the large peripheral cells of the brain and myotube system. The myotube system is composed of multiple myoblasts and is characterized by multinucleation. The large cells at the brain periphery gradually become distinguishable from the myofibroblasts. There were also white adipocytes, blood vessels surrounded by multiple large cells, and pigment cells that could be clearly seen in the peripheral brain tissue. Pigment cells exhibit a protective function similar to the skull and blood–brain barrier. Some of the cells observed at the brain periphery were not of the traditional round shape but rather were fibrous (Figure 1D). Some cells in the brain can also extend to the peripheral tissue of the brain, and can communicate with each other, rather than existing independently.

**FIGURE 2:**
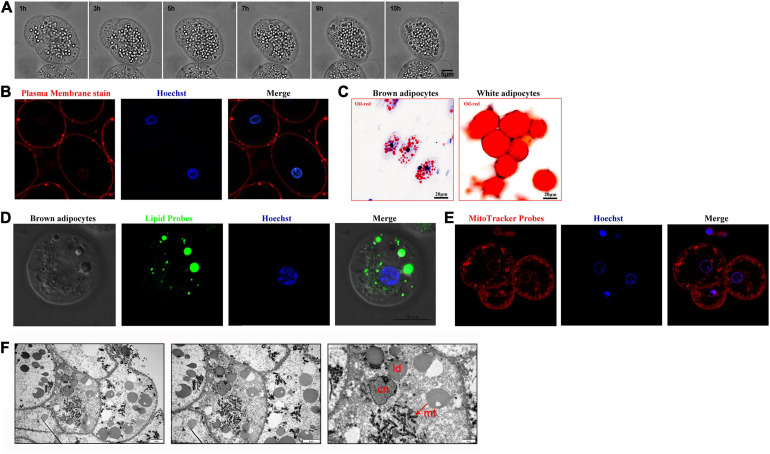
Morphological characteristics of brown adipocytes surrounding the brain tissue of lampreys. **(A)** Time-lapse images of brown adipocytes were acquired every 10 min for 24 h. **(B)** The brown adipocytes were incubated in medium containing 0.5 μg CellMask^TM^ orange plasma membrane stain for 3 min and then stained with Hoechst. **(C)** Oil Red O was used to examine the morphology of brown adipocytes and white adipocytes. **(D)** Brown adipocytes were stained with lipid probes prior to staining with Hoechst. **(E)** The brown adipocytes were incubated in medium containing 30 nM MitoTracker Red. Merged images of cells double-stained with Hoechst are shown. **(F)** Mitochondria with dense cristae and larger lipid droplets in brown adipocytes under transmission electron microscopy (left pane). A higher magnification image is shown in the right pane.

The morphological characteristics of the large cells were observed by live cell imaging microscopy and confocal fluorescence microscopy. The results demonstrated that the morphology of the cells was round or oval and that they were approximately 30–50 μm in diameter; the cytoplasm contained multiple dispersed lipid droplets, and the round nucleus was located in the cell center (Figures 2A,B). When cells were stained with Oil Red O and lipid probes, the results were consistent with those described above (Figures 2C,D). In addition, a large number of mitochondria were distributed in the inner membrane of cells according to either mitochondrial staining combined with confocal fluorescence microscopy or transmission electron microscopy (Figures 2E,F). In summary, we referred to the large cells as multilocular adipocytes.

### Identification of the Molecular Features and Functional Phenotypes of Brown-Like Adipocytes in Multilocular Adipocytes

**FIGURE 3:**
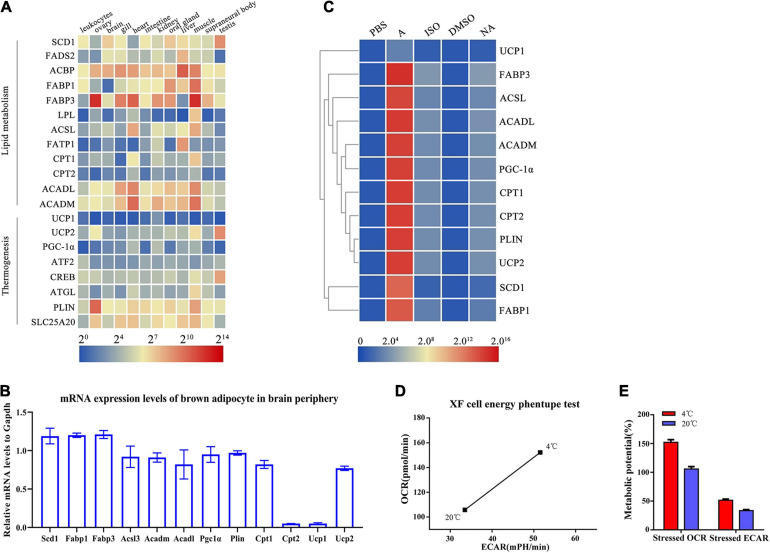
Identification of lamprey brown adipocytes through molecular and functional analysis. **(A)** Expression profiles of brown adipocyte-enriched markers and key transcription factors in lamprey tissues obtained by real-time PCR. Gapdh expression was used as an internal control. The expression profiles of marker genes are shown in a heat map. Blue and red indicate low and high expression levels. **(B)** The mRNA expression of selected genes enriched in lamprey brown adipocytes. **(C)** The lamprey brown adipocytes were treated with adrenaline (1 μM), isoprenaline (10 μM) and norepinephrine (0.5 μM) for 24 h at 18°C. Expression analysis of enriched markers and key transcription factors was performed using real-time PCR. All of the data are presented as the means ± SDs based on three independent cDNA samples with three replicates per sample. **(D)** Metabolic profile of the stimulatory effect of temperature on oxidative phosphorylation and glycolysis. **(E)** The stressed OCRs and ECARs of glycolysis and oxidative phosphorylation. Each bar represents the mean value from three determinations with the standard deviation (SD).

To further identify gene signatures and characterize the thermogenic potential of the cells surrounding the brain in lampreys, we conducted RNA-seq analysis of the multilocular adipocytes and annotated all genes based on the first chromosomal-level assembly of the Reissner lamprey genome (PRJNA558325). Many homologs of thermogenesis and lipid metabolism regulators found in human brown adipocytes were identified in lampreys, as shown in Table 1. The mRNA expression of these genes was detected in different lamprey tissues. Our data demonstrated that Acbp was highly expressed in all tissues, while Scd1, Fads2, Fabp1, Fabp3, Acadl, Acadm, Plin and Slc25a20 were highly expressed in the gill, kidney, muscle and supraneural body (Figure 3A). Interestingly, the expression of Ucp1, which is considered to be related to thermogenesis in mammals, was very low in all tissues of lampreys. We also detected the expression of BAT metabolism and thermogenic regulators, such as Scd1, Fabp1, Fabp3, Acsl, Acadm, Acadl, Pgc-1α, Plin, Cpt1, Cpt2 and Ucp1, in lamprey multilocular adipose cells. The results showed that the mRNAs of Scd1, Fabp1, Fabp3, Acsl3, Acadm, Acadl, Pgc-1α, Plin and Cpt1 were expressed in the multilocular adipose cells, while Cpt1 and Ucp1 showed extremely low expression, and the mRNA expression of Ucp2 was higher than that of Ucp1 (Figure 3B).

**TABLE 1:** Identification of the molecule relative to thermogenesis and lipid metabolism in lamprey.

Gene symbol	Description	ORF (bp)	Amino acids (aa)	Function	References
SCD1	Stearoyl-CoA desaturase 1	1140	379	It plays a pivotal role in the synthesis of fatty acids.	Young et al. (2010)
FADS2	Fatty acid desaturase-2	1143	380	It plays a very important role in the biosynthesis of polyunsaturated fatty acids.	Kanehisa et al. (2008)
ACBP	Acyl coenzyme A binding protein	264	87	It can bind, store and transport long chain lipophyl CoA with high specificity and affinity.	Mao et al. (2005)
FABP1	Fatty acid-binding protein 1	396	131	It regulates fatty acid oxidation, lipid metabolism such as triglyceride synthesis and phospholipid synthesis.	Shu et al. (1999)
FABP3	Fatty acid-binding protein 3	405	134	It mainly binds free fatty acids and other hydrophobic ligands in cells and transports them to organelles such as peroxidase mitochondria and nucleus.	Gesta et al. (2007)
LPL	Lipoprotein lipase	1467	488	It can catalyze the hydrolysis of triglyceride to glycerol and free fatty acid.	Rodeheffer et al. (2008)
ACSL	Long-chain fatty acyl CoA synthetases	1413	470	It catalyzes the synthesis of substrate fatty acyl coenzyme A from fatty acids and coenzyme A.	Ohno et al. (2012)
FATP1	Fatty acid transport protein 1	1425	474	It is an important protein for the transport of long chain fatty acids.	Kajimura et al. (2009)
CPT1	Carnitine palmitoyltransferase 1	2076	691	It is a key enzyme for carnitine-dependent transport through the mitochondrial inner membrane	Mendes et al. (2020)
CPT2	Carnitine palmitoyltransferase 2	2091	696	It can oxidize long-chain fatty acids in the mitochondria.	Blondin et al. (2020)
ACADL	Acyl-CoA dehydrogenase long chain	1683	560	It is a family of mitochondrial flavin enzymes involved in the metabolism of fatty acids and branched amino acids.	Zhao et al. (2020)
ACADM	Acyl-CoA dehydrogenase medium chain	1353	450	It can catalyze the initial steps of mitochondrial fatty acid-oxidation pathway.	Kim et al. (2006)
UCP1	Uncoupling protein 1	951	316	This gene is expressed only in brown adipose tissue, a specialized tissue which functions to produce heat.	Ricquier (2017)
UCP2	Uncoupling protein 2	957	318	It is thought to play a role in non-shivering thermogenesis, obesity and diabetes.	Li et al. (2019)
PGC-1α	PPARγ coactivator 1 Alpha	2244	747	The protein encoded by this gene is a transcriptional coactivator that regulates the genes involved in energy metabolism.	Cheng et al. (2018)
ATF2	Cyclic AMP-dependent transcription factor	1593	530	It is critical for induction of thermogenic genes by cAMP in BAT.	Yoon et al. (2018)
CREB	CAMP responsive element binding protein	690	229	An important transcription factor regulating adipocyte differentiation.	Berdeaux and Hutchins (2019)
ATGL	Adipose triglyceride lipase	2259	752	It can catalyze the first step in the hydrolysis of triglycerides in adipose tissue.	Sathyanarayan et al. (2017)
PLIN	Perilipin	1494	497	The protein encoded by this gene coats lipid storage droplets in adipocytes, thereby protecting them until they can be broken down by hormone-sensitive lipase.	Hayward et al. (2017)
SLC25A20	Solute carrier family 25 member 20	903	300	This protein mediates the transport of acylcarnitines into mitochondrial matrix for their oxidation by the mitochondrial fatty acid-oxidation pathway.	Iacobazzi et al. (2004)

Because the multilocular adipose cells showed some morphological and molecular characteristics of BAT, we suspected that these cells might be thermogenic and exhibit a function related to energy metabolism. To further characterize the thermogenesis potential of multilocular adipose cells, the cells were treated with adrenaline, isoprenaline and noradrenaline, which are thermogenesis-inducing agents in mammalian BAT. We compared the gene expression levels of these thermogenic and lipid metabolism regulators in multilocular adipose cells before or after stimulation. The expression of these important regulators was significantly higher in multilocular adipose cells treated with adrenaline than in control cells treated with PBS (Figure 3C). However, there was a little change in the expression of these genes when multilocular adipose cells were treated with isoprenaline and noradrenaline (Figure 3C). In addition, we found that Ucp1 did not respond to stimulation; however, the expression of Ucp2 was highly upregulated after treatment with adrenaline.

The Seahorse XF Extracellular Flux Analyzer provides real-time measurements of mitochondrial respiration and glycolysis in cultured cells, reported as OCR and ECAR values, respectively. Bioenergetic assays of multilocular adipocytes showed an altered mitochondrial function at 4°C relative to 20°C. The basal OCR- and ECAR-induced maximal OCR of multilocular adipose cells showed an approximately 50% increase at 4°C compared to that at 20°C (Figure 3D). The multilocular adipose cells treated at 4°C showed increases in both OCR and ECAR relative to those in the control at 20°C. The stressed OCR (%) at 4°C was higher than that in the control, but the difference was not significantly greater than that for the stressed ECAR (%) (Figure 3E). This finding indicated that treatment at 4°C preferentially changed the energy production route from aerobic to glycolytic metabolism. In conclusion, we preliminarily considered the multilocular adipocytes in the periphery of the lamprey brain to be brown-like adipocytes.

### Molecular Evolution of the UCP Gene Family, a Characteristic Marker of Brown Adipocytes

**FIGURE 4:**
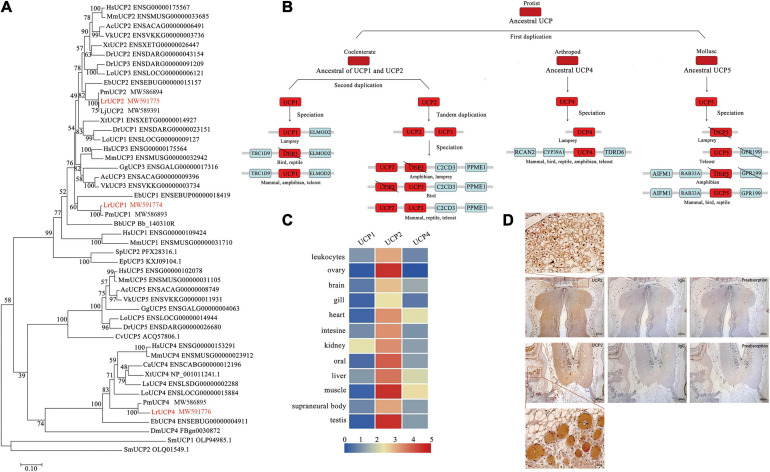
Phylogenetic analysis and expression profiling of UCPs in lampreys. **(A)** Phylogenetic tree of the UCP family based on the NJ method. The numbers on interior branches indicate the bootstrap values derived from 1000 replications. All the amino acid sequence data used are from the Ensemble database and NCBI database. Hs, *Homo sapiens*; Mm, *Mus musculus*; Gg, *Gallus gallus*; Ls, *Lonchura striata*; Ac, *Anolis carolinensis*; Vk, *Varanus komodoensis*; Ca, *Chelonoidis abingdonii*; Xt, *Xenopus tropicalis*; Dr, *Danio rerio*; Lo, *Lepisosteus oculatus*; Eb, *Eptatretus burgeri*; Lr, *Lethenteron reissneri*; Bb, *Branchiostoma belcheri*; Dm, *Drosophila melanogaster*; Cv, *Crassostrea virginica*; Ml, *Macrostomum lignano*; Sm, *Symbiodinium microadriaticum*. **(B)** Evolutionary scenario of the UCP gene in vertebrates. The major evolutionary events are highlighted. The \ symbol indicates genes that have been lost. **(C)** Expression of UCPs in different tissues of Reissner lampreys. **(D)** Immunohistochemistry of LrUCP2 in brown adipocytes in lamprey brains.

UCP1 is an important marker molecule of brown adipocytes. By searching the Reissner lamprey genome database, we identified three UCP gene families. Based on structural domain, motif and syntenic gene analyses, three UCP genes were identified as orthologous genes of mammalian UCP1, UCP2 and UCP4 (Supplementary Figure 1). The phylogenetic analysis showed that the UCP paralogs of Reissner lamprey can be grouped into two major clades: one encompassing the LrUCP1 and LrUCP2 paralogs and another comprising the LrUCP4 paralog. The results also showed that LrUCP1 appears to be an ancestral molecule of vertebrate UCP1, UCP2, and UCP3, whereas UCP2 and UCP4 show high homology with other vertebrate homologs (Figure 4A). From the above results, we deduced the evolutionary history of the UCP gene family (Figure 4B). The origins of the UCP molecule can be traced back to protists. The analyses suggest that UCPs were acquired through three rounds of gene duplication, in which the first duplication gave rise to the ancestral UCP1-UCP3, UCP4 and UCP5 genes. The second duplication gave rise to the ancestral UCP1 and UCP2/UCP3 genes, and the third round of duplication, which gave rise to UCP2 and UCP3, occurred early in invertebrate evolution. Subsequent to these duplications, different UCP genes were lost in different lineages. LrUCP3 and LrUCP5 were lost after the duplication event that occurred after lampreys differentiated from other vertebrates.

In addition, an expression profiling analysis of the lamprey UCP gene family was conducted. By comparing the expression of the three genes in lamprey tissues, it was demonstrated that UCP2 was more widely and highly expressed in different tissues than UCP1 and UCP4 (Figure 4C). Considering these findings together with the above analysis and the practical results, we conclude that UCP2 is likely to be a molecule that plays a major role in the Reissner lamprey. A prokaryotic expression vector of LrUCP2 was subsequently constructed, and the LrUCP2 recombinant protein was purified to prepare a mouse antibody (Supplementary Figure 2). Immunohistochemistry was used to detect the expression of UCP2 in brown-like adipocytes, and it was found that UCP2 was widely and highly expressed in these cells and showed an intracellular dotted distribution (Figure 4D).

### The Effect of Lamprey UCP2 on Differentiation Into Brown Adipocytes and Decreases in Intracellular ATP and FFA Contents

**FIGURE 5:**
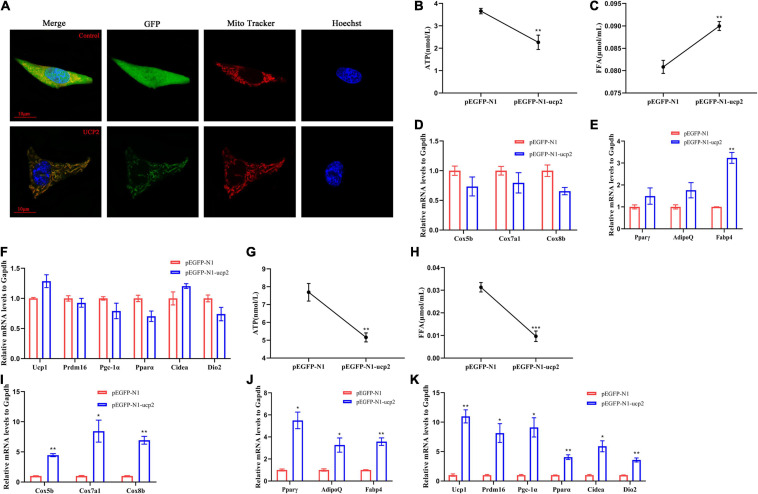
Localization and functional identification of lamprey UCP2 in 3T3L1 cells. **(A)** The localization of lamprey UCP2 in 3T3L1 cells was observed by confocal microscopy at 30 h after UCP2 overexpression in 3T3L1 cells. **(B,C)** ATP content and FFA content of 3T3L1 cells overexpressing UCP2. **(D–F)** mRNA levels of mitochondrial genes **(D)**, general adipocyte markers **(E)**, and brown-selective genes **(F)** after overexpression of UCP2 in 3T3L1 cells (*n* = 3). **(G,H)** ATP content and FFA content of mature brown adipocytes overexpressing UCP2. **(I–K)** mRNA levels of mitochondrial genes **(I)**, general adipocyte markers **(J)**, and brown-selective genes **(K)** in mature brown adipocytes overexpressing UCP2 (*n* = 3). All expression data are presented as the mean ± SD; *P < 0.05; **P < 0.01; ***P < 0.001.

To further describe the important role of UCP2 in brown-like adipocytes at the periphery of the lamprey brain, mouse 3T3-L1 were used, and LrUCP2 was inserted into the pEGFP-N1 eukaryotic expression vector. The results obtained by confocal laser imaging showed that LrUCP2 was located on the mitochondria of 3T3-L1 cells (Figure 5A). To demonstrate the function of UCP2 more accurately, G418 was used to screen the cell lines with stable UCP2 expression (Supplementary Figure 3). When LrUCP2 was overexpressed in 3T3-L1 cells, the intracellular ATP content was decreased, and the FFA content was increased compared to those in the control (Figures 5B,C). The mRNA expression levels of mitochondrial proteins and brown adipocyte marker molecules did not change; however, the mRNA levels of Fabp4 were upregulated (Figures 5D–F). When the 3T3-L1 cells overexpressing LrUCP2 were differentiated, both the intracellular ATP and FFA contents were decreased compared to those in the control (Figures 5G,H), and the mRNA expression levels of mitochondrial proteins, adipocyte differentiation and brown adipocyte marker molecules were all increased (Figures 5I–K). These results suggest that LrUCP2 has different functions in 3T3-L1 cells and differentiated 3T3-L1 cells. The overexpression of LrUCP2 can upregulate the expression of Fabp4 and increase the intracellular FFA content in 3T3-L1 cells, which may promote the decomposition of lipids and the transport of fatty acids. In contrast, the overexpression of LrUCP2 in differentiated 3T3-L1 cells may accelerate browning.

### The Global Transcriptome of Brown-Like Adipocytes Reveals the Role of the Immune Response

**FIGURE 6:**
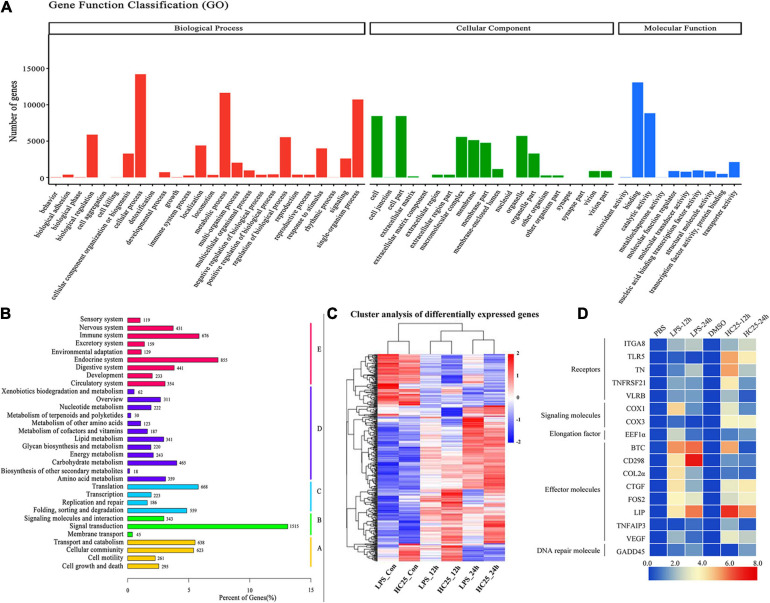
Transcriptome analysis of brown adipose cells at the periphery of the lamprey brain. **(A)** GO enrichment map of all the annotated unigenes, which were divided into three categories. **(B)** Kyoto Encyclopedia of Genes and Genomes (KEGG) classifications of non-redundant unigenes. A, cellular processes; B, environmental information processing; C, genetic information processing; D, metabolism; E, organismal systems. **(C)** Global transcriptome landscape of lamprey brown adipocytes treated with PBS, DMSO, LPS (100 μg/mL) and 25HC (50 μM). The processing time was 12 h or 24 h. Heatmap of clustered correlations among the six groups. **(D)** Heatmap of immune molecule expression levels, which were determined via Q-PCR in lamprey brown adipocytes after stimulation with PBS, DMSO, LPS (100 μg/mL) and 25HC (50 μM) at 12 h and 24 h. Blue and red represent low and high expression levels, and the color scales correspond to the expression levels of the genes.

A previous study demonstrated that lampreys exhibit a blood–brain barrier, similar to other vertebrates, and an endothelial blood–brain barrier to macromolecules. However, the five brain regions of lampreys, surrounded by a layer of brown-like adipocytes, are exposed in the brain cavity, and it is unclear whether the layer of brown-like adipocytes is involved in the immune response against pathogens. To characterize the immune response of brown adipocytes from a molecular perspective, we performed paired-end RNA-seq analysis of poly(A)-selected RNAs from brown-like adipocytes responding to LPS and 25HC treatments. We firstly calculated all possible pairwise correlations among the four samples by using the expression levels of annotated protein-coding genes to quantitatively reveal the overall transcriptome proximity of normal brown-like fat cells to that of the treatment group. All unigenes were assigned to three GO term categories: biological processes, cellular components, and molecular functions (Figure 6A). According to the KEGG classification of all unigenes, 11,324 genes were mapped to 35 different pathways. Among these pathways, “signal transduction” (1,515; 13.4%), “endocrine system” (855; 7.6%) and “immune system” (676; 6.0%) were the most prominent pathways (Figure 6B). The differential expression data revealed many gene expression differences between brown-like adipocytes subjected to LPS/25HC treatment and those not receiving LPS/25HC-treatment, showing a more than 2-fold difference in mRNA transcript levels (Figure 6C). Thus, these data demonstrated the upregulation of energy expenditure pathways and repression of inflammation in normal brown adipocytes relative to those subjected to LPS and 25HC treatment. The expression levels of immune molecules were further analyzed via real-time PCR using total RNA obtained from LPS/25HC-treated or untreated brown-like adipocytes (Figure 6D and Supplementary Table 1). The real-time PCR results were consistent with those of the transcription analysis. Taken together, the brown-like adipocytes are not only a distinct fat depot but also function as immune cells with a role in immune defense.

## Discussion

Jawless vertebrates are the most primitive vertebrates on earth and appeared 520 million years ago, as early as the Cambrian period. In 1999, a paper in Nature reported that fossils of Myllokunmingia and Halou:Chthys were found among the early Cambrian Chengjiang Fauna in China (Shu et al., 1999). These two fish have spindle-shaped bodies, W-shaped sarcomeres, and relatively complex bony skulls, gill arches, pericardial cavities, and fin bars. These characteristics are similar to those of current lamprey larvae, which represent the origin of the vertebrates.

Although ostracoderm fossils have been found as far back as the Middle Ordovician and even the late Cambrian, the most promising vertebrates had not fully developed at that time, and the whole Ordovician ocean was still the realm of invertebrates. At the end of the Ordovician period, due to the influence of the great ice age of the Hirnantian, vertebrates suffered mass extinction, and vast ecosystems emerged in the ancient oceans. The ostracoderms that survived the great ice age gave rise to radiant development during the Silurian-Devonian period. The agnathans passed through the late Silurian and Early Devonian and began to decline in the Middle Devonian. Finally, nearly all of the agnathan fishes disappeared because of the late Devonian extinction, with the exception of lampreys and hagfish. Thus, the question of why lampreys have survived the long evolutionary process arises. The reason is closely related to their unique way of life and genetic characteristics. In this study, we identified a type of tissue surrounding the lamprey brain that is composed of a series of brown-like adipocytes, and the function of these cells in lampreys may provide some clues to answer the above question.

In mammals, there are two types of brown adipocytes: classic brown adipocytes in rodents, which appear in scapular tissues, and brown-like adipocytes or brite adipocytes, identified in WAT in specific situations. The former cell type is formed by the differentiation of brown precursor adipocytes, while the latter may originate from stem cells that are derived from adipose tissue or are directly transformed from white adipocytes (Gesta et al., 2007; Rodeheffer et al., 2008; Seale et al., 2008; Ohno et al., 2012). Classic brown adipocytes arise from Myf5-positive myofibroblast precursors, and the coexpression of C/EBPβ and PRDM16, which controls the differentiation of precursor cells into brown adipocytes, induces myofibroblast differentiation into brown adipocytes (Kajimura et al., 2009). The multilocular adipocytes that we found at the periphery of the brain become distinguishable from the myofibroblasts; these brown-like adipocytes may differentiate from myofibroblasts, but further experimental investigation is required.

Lamprey adipose tissue contains a range of transcription factors and important regulators similar to those of mammals, such as Pgc-1α and Ucp1. The thermogenesis of brown-like adipocytes is related to the regulation of epinephrine in lampreys. β–Adrenergic activators stimulate beige and brown adipocyte development in mammals (brown and beige fat). In addition, Ucp1 shows almost no change after stimulation and is poorly expressed in various tissues, whereas Ucp2 shows high expression and a thermogenic response, suggesting that Ucp2 is likely to be the molecule that has more important functions in lampreys. Studies have shown that mammalian orthologs display an accelerated evolutionary rate relative to non-mammalian Ucp1 orthologs and other mammalian paralogs, and the thermogenic function of Ucp1 in placental mammals seems to result from such neofunctionalization (Mendes et al., 2020). The immunohistochemical results showed that UCP2 was highly expressed in peripheral multilocular adipocytes of the lamprey brain. Thus, we consider the brown-like adipocytes found in adult lampreys to be a unique type of primitive thermogenic adipose tissue. Interestingly, we found that the lamprey β-adrenergic receptor shows the highest similarity to β1 adrenergic receptors in humans and mice, however, the molecules that receive thermogenesis signals in humans and mice are β2 and β3 adrenergic receptors (Blondin et al., 2020). This may explain why these brown-like adipocytes in lampreys respond very strongly only to adrenaline.

Brown adipose tissue in mammals burns fatty acids and generates heat by uncoupling oxidative phosphorylation from ATP production in mitochondria. However, birds produce heat through their muscles and regulate their body temperature. Several species of fish maintain brain temperatures that are higher than the surrounding environmental temperature through ATP-dependent Ca^2+^ cycling in the sarcoplasmic reticulum of “heating cells” around the brain. The present study showed that the brown-like adipocytes around the lamprey brain contain “heating cells” similar to those of other fish. As shown in Figures 3D,E, the treatment of multilocular adipose cells at 4°C increased both ECAR level and OCR compared to those in the control at 20°C, and the 4°C treatment preferentially changed the energy production route from aerobic to glycolytic metabolism to produce heat to maintain basal metabolism.

According to our results, we propose that LrUCP2 is involved in regulating brown-like adipocyte differentiation. The expression of brown adipocyte markers, mitochondrial proteins and adipocyte differentiation markers was upregulated in differentiated 3T3-L1 cells which overexpressing UCP2. Our results indicate that LrUCP2 overexpression can induce positive feedback regulation of mouse UCP1 and its upstream regulatory factors in 3T3-L1 cells, and the precise regulatory mechanism needs further experimental verification.

Furthermore, our transcriptome results shed light on the potential immune activity of lamprey multilocular fat cells that may affect metabolic events in these cells. Some DEGs related to immune pathways were identified in lamprey adipocytes after LPS and 25HC treatment. According to the DEG annotation information, we found upregulation of proinflammatory factors that play a role in resisting external stimuli, while the expression of some molecules associated with lipid metabolism was downregulated. There may be a complex regulatory network between the inflammatory response and metabolic function in adipose cells.

Based on the above results, we considered the cells surrounding the lamprey brain not only be equivalent to the brown adipocytes of higher species but also to exhibit unique functional characteristics. The brown-like adipose tissue that cocoons the lamprey brain protects the structure of its five parts, and these cells may provide material energy for adaptation to long fasting periods during the lifecycle. These brown-like adipocytes can consume energy and generate additional heat to maintain a sufficient temperature for lampreys to complete their lifecycle in benthic and cold water environments and to ensure normal physiological function and developmental processes. Chung-Davidson also revealed a secondary sexual characteristic of male sea lamprey to be thermogenic adipose tissue that instantly increases heat production during sexual encounters. This tissue is located in front of the anterior dorsal fin of mature males and presents as a swollen dorsal ridge, called rope tissue, but is not BAT (Chung-Davidson et al., 2013). In addition, our results showed that the brown-like adipose tissue around the brain may function similarly to the blood–brain barrier of higher vertebrates and protect against pathogen invasion. Although lampreys do not form a blood–brain barrier and show incomplete development of the adaptive immune system, the protective mechanisms of these animals and their defense responses against pathogens are unique in nature. Hence, further insight into the mechanisms in the lamprey brain directing adaptation to the environment and responses that support the survival of the organism is needed.

In conclusion, we identified brown-like adipocytes around the lamprey brain. These cells may provide nutrients and energy sources supporting metabolic activity in the brain and protect the brain from outside pathogen infection. The identification of brown-like adipocytes in the lamprey brain has improved our understanding of the mechanism of thermogenesis in mammals and the unique characteristics of lampreys. Further investigations of the significance and specificity of this kind of cell with thermogenic potential in lampreys and the related regulatory pathways are an important future goal.

## Data Availability Statement

The datasets presented in this study can be found in online repositories. The names of the repository/repositories and accession number(s) can be found below: NCBI, PRJNA733594.

## Ethics Statement

The animal study was reviewed and approved by Animal Welfare and Research Ethics Committee of the Institute of Dalian Medical University. Written informed consent was obtained from the owners for the participation of their animals in this study.

## Author Contributions

YP, XX, and QL wrote the main manuscript, designed the experiments, reviewed the study results, and revised the manuscript. XW and WC prepared Figure 1. AM and XX prepared Figures 2, 6. YP, XX, and TL prepared Figures 3–5 and the Supplementary Figures. JL provide the lamprey material. All authors read, contributed to, and approved the final manuscript.

## Conflict of Interest

The authors declare that the research was conducted in the absence of any commercial or financial relationships that could be construed as a potential conflict of interest.

## Footnotes

1 http://www.genome.jp/kegg/

## Supplementary Material

Supplementary Figure 1 The evolutionary analysis of UCPs in lamprey. **(A)** The domains of UCPs from different species. The Mito_carr (mitochondrial carrier protein) domains are shown with orange boxes based on identification by Pfam. **(B)** The MEME motifs and sequence alignment of the UCP family. A total of 26 motifs are shown in different colored squares. **(C)** The red boxes in the sequence alignment indicate the three energy transfer protein signatures. **(D)** Conservation of genes neighboring UCP1, UCP2, and UCP4. The orthologous genes were similarly color-coded, and arrows or arrowheads in opposite directions indicate genes located on opposite strands.

Supplementary Figure 2 Preparation of the recombinant UCP2 protein and polyclonal antibody. **(A)** Analysis of LrUCP2 expression via SDS-PAGE. M, low molecular-weight protein marker. The LrUCP2 protein concentration is 0.4 mg/mL. **(B)** Antibody titer of anti-LrUCP2 antibodies. **(C)** Western blot of BAT in the peripheral of brain and the rL-UCP2 protein in lampreys.

Supplementary Figure 3 Screening for cell lines with stable UCP2 expression. Cells with stable expression of UCP2 and its control were obtained after 10 days of screening.
