# Supplementary figures and images for: Genetic and Functional Characterization of Novel Brown-Like Adipocytes Around the Lamprey Brain
Source: Front Cell Dev Biol. 2021 Jul 1;9:674939. doi: 10.3389/fcell.2021.674939 (PMC8281276; doi:10.3389/fcell.2021.674939)

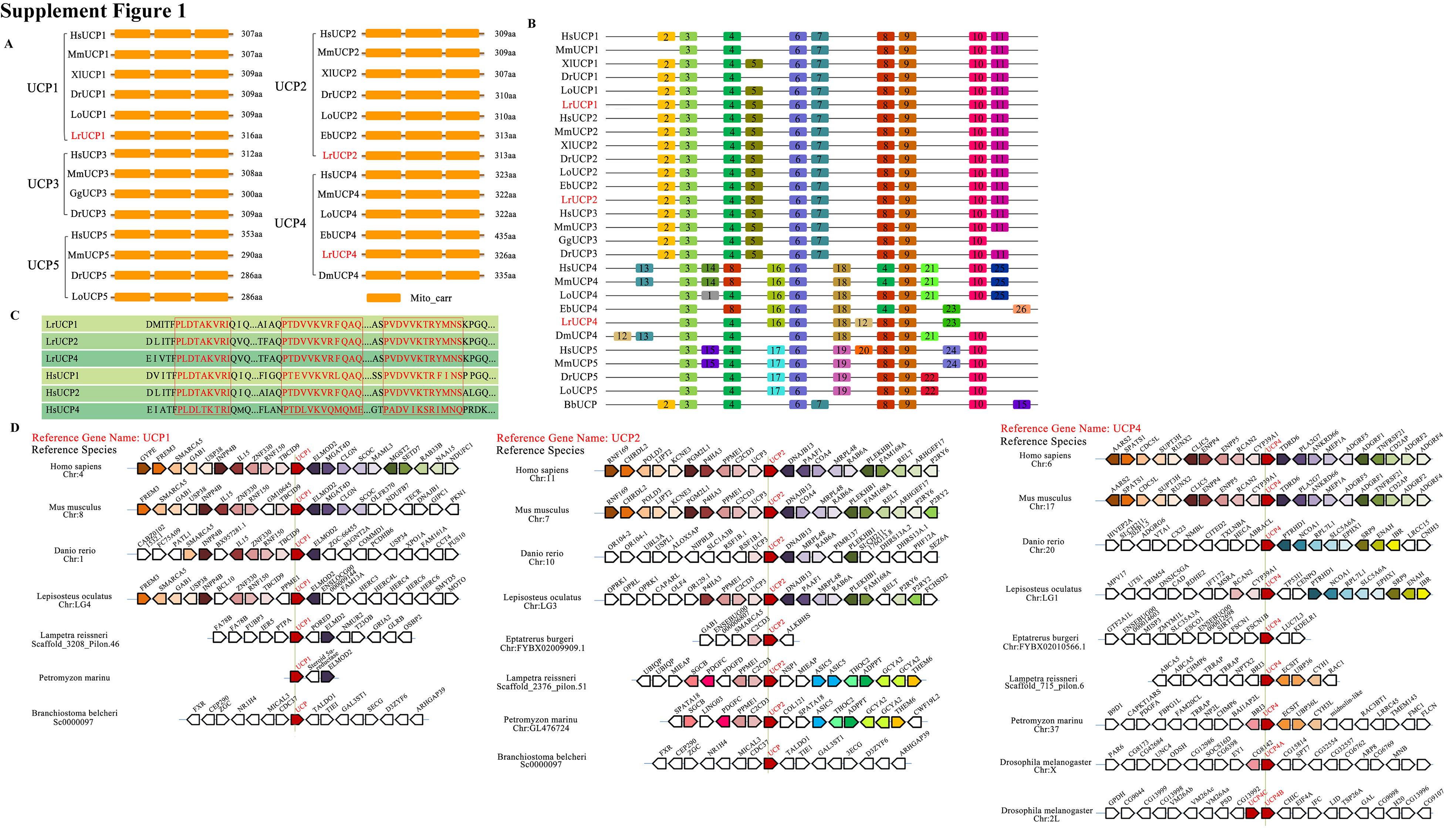

Supplement: Supplementary Figure 1 — The evolutionary analysis of UCPs in lamprey. (A) The domains of UCPs from different species. The Mito_carr (mitochondrial carrier protein) domains are shown with orange boxes based on identification by Pfam. (B) The MEME motifs and sequence alignment of the UCP family. A total of 26 motifs are shown in different colored squares. (C) The red boxes in the sequence alignment indicate the three energy transfer protein signatures. (D) Conservation of genes neighboring UCP1, UCP2, and UCP4. The orthologous genes were similarly color-coded, and arrows or arrowheads in opposite directions indicate genes located on opposite strands. [file Image_1.TIF]

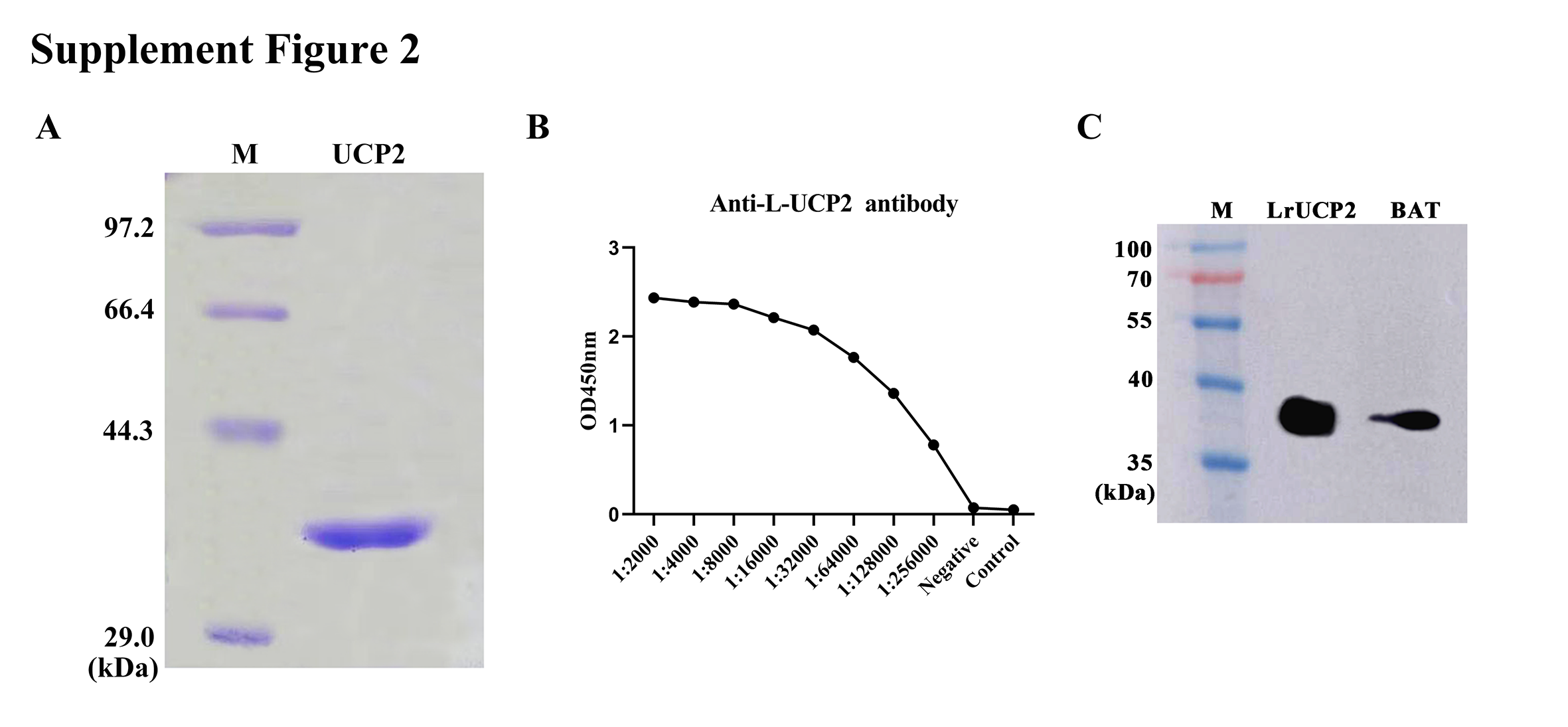

Supplement: Supplementary Figure 2 — Preparation of the recombinant UCP2 protein and polyclonal antibody. (A) Analysis of LrUCP2 expression via SDS-PAGE. M, low molecular-weight protein marker. The LrUCP2 protein concentration is 0.4 mg/mL. (B) Antibody titer of anti-LrUCP2 antibodies. (C) Western blot of BAT in the peripheral of brain and the rL-UCP2 protein in lampreys. [file Image_2.TIF]

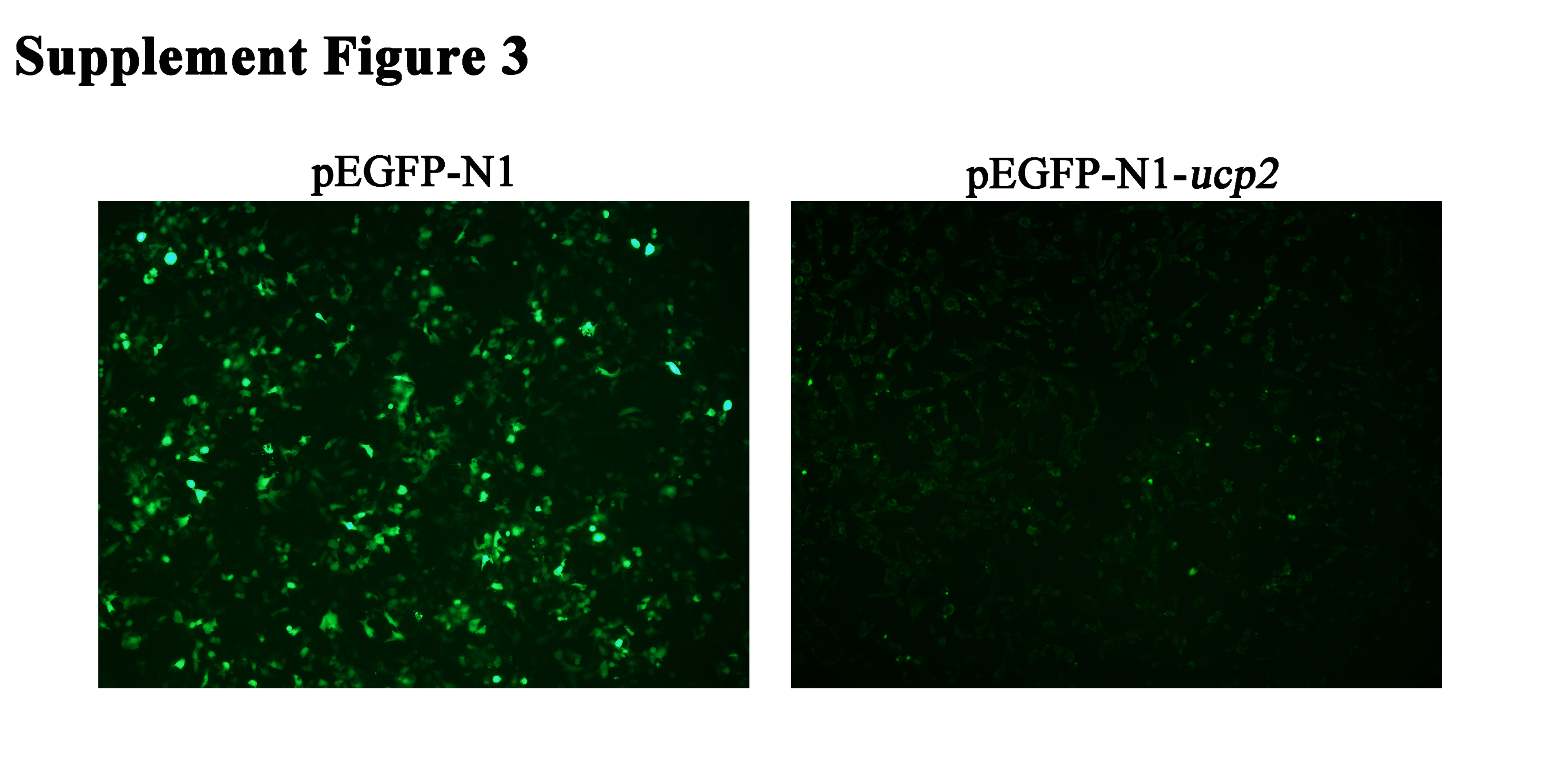

Supplement: Supplementary Figure 3 — Screening for cell lines with stable UCP2 expression. Cells with stable expression of UCP2 and its control were obtained after 10 days of screening. [file Image_3.TIF]
