# Supplementary material for: Genetic and Functional Characterization of Novel Brown-Like Adipocytes Around the Lamprey Brain
Source: Front Cell Dev Biol. 2021 Jul 1;9:674939. doi: 10.3389/fcell.2021.674939 (PMC8281276; doi:10.3389/fcell.2021.674939)
Supplement: Supplementary file 4 [file Data_Sheet_1.docx]

**Supplement Table 1. Identification of the immune molecule in lamprey.**

| Gene symbol | Description | ORF(bp) | Amino Acids(aa) |
| --- | --- | --- | --- |
| BTC | probetacellulin | 942 | 313 |
| CD298 | potassium-transporting ATPase subunit beta-1 | 1011 | 336 |
| COL2α | collagen type II alpha 1 chain | 4431 | 1476 |
| COX3 | cytochrome c oxidase subunit III | 405 | 134 |
| CTGF | connective tissue growth factor | 1200 | 399 |
| EEF1α | elongation factor 1-alpha 1 | 255 | 84 |
| FOS2 | fos-related antigen 2 isoform X1 | 915 | 304 |
| GADD45 | growth arrest and DNA damage-inducible protein GADD45 beta | 729 | 242 |
| ITGA8 | integrin alpha-8 | 3171 | 1056 |
| TN | tenascin isoform X8 | 2352 | 783 |
| TNFAIP3 | TNFAIP3-interacting protein 1 | 693 | 230 |
| VEGF | vascular endothelial growth factor | 615 | 204 |
| VLRB | variable lymphocyte receptor B | 822 | 273 |
| LIP | lamprey immune protein | 942 | 313 |
| TLR5 | toll-like receptor 5 | 2592 | 863 |
| TNFRSF21 | tumor necrosis factor receptor superfamily member 21 | 2595 | 864 |
| FOSB | fos-related antigen | 780 | 259 |
| COX1 | cytochrome c oxidase subunit I | 543 | 180 |

**Supplement Table 2. The sequences of primers used for real-time PCR analysis in lamprey.**

| Gene symbol | Accession number | 5’ Primer Sequence | 3’ Primer Sequence |
| --- | --- | --- | --- |
| BTC | MW600637 | CTACAGCGACGACGACGAAGAAC | CGCCGCTCATCTCGTAGTCAAC |
| CD298 | MW600638 | TTCCGCGCCGAGATCTACTACTC | CGTCTGCTTCTCGTTGCTGTAGG |
| COL2α | MW600639 | AGGAGGATTCGACGAGAAGTCAGG | CTTGCTCTCCACGGATGCCTTG |
| COX1 | MW600652 | CCCTAGACCAAACCTACGCC | CGAGTAACGTCGGGGCATTC |
| COX3 | MW600640 | CACTTCCACTCCATAACGCTCCTC | CGTGTTACATCGCGCCATCATTG |
| GADD45 | MW600644 | TTCTGCGGCGACAACGACATC | TGCTGGTGGTGGTGGTGGAG |
| VEGF | MW600648 | CAGCAGCAACAGCAGCAACAAC | CACGACGAGGAAGAGCACGAAC |
| CTGF | MW600641 | CCGAGCCAAGGAATGCGAACTC | CAGCGGCAGACATTGGAGCAC |
| FOS2 | MW600643 | AGCCTCATCATCATCAGCAGCAAC | CTCTCCGCTTCTCGTCCTCCTC |
| ITGA8 | MW600645 | ACCACTACCAGGCAGACCACATC | GGTTGGAGCAGTTGAGCGTGAG |
| TNFAIP3 | MW600647 | GAAGCAGAACAGCCAGAAGACCTC | CGGCATCACTGTCGCTCTCAC |
| EEF1α | MW600642 | CGCCGCTCTTGAGGCTCTTG | ACATCGCGTGCAAGTTCTCAGAG |
| TN | MW600646 | ACAGCGTCCACAAGAAGCAGTTG | TGCCTTGCCGTCAATCACAGATG |
| TNFRSF21 | MN395391 | CCCATTGAGCAGGTTGGTTT | GCCGAGTTGTCGGAAGTTCTA |
| LIP | MW630112 | TGGCATCTGCTTGGGTGTT | CACGTTAGGCAGAATTTGGT |
| VLRB | MW600649 | TCGCTCCCCAACACTCTCA | TCCACTTGATCCACATGATGGT |
| TLR5 | MW600650 | TGGACCTGAGCTTCAACATGAT | AAGTTTCGGCTCAGGTCGAGT |
| SCD1 | MW588007 | CGAGACCAGCAGCAGCAACG | ATCGTCCTCCGTCGCCTTGG |
| FABP1 | MW588008 | TCGGAAACATGACCAAACCCACAC | CATCCGCAGTCGTCTCCTTGAAC |
| FABP3 | MW588009 | GGAGTCGCACAGCACCTTCAAG | TCTTCCCGTCCACCGTCAGC |
| ACSL | MW588010 | CGATGGCAGAGGTAGCAATGTGG | CGCAAGAAGAGACGCAAGGAGAC |
| ACADL | MW588011 | CCTCCGCATCTTCCGCATCTTC | TTGCCCGCACCCTGGATACC |
| ACADM | MW588012 | ATGGTGGCAAGGCAAACTGGTAC | ACTCCTGGTGTGTCGGCATCC |
| PGC-1α | MW588013 | GGTCGATTCGGCCTTAAATG | GTTCCCGTCGCCAACTCA |
| PLIN | MW588014 | AAATTGCTGCTGATGCTGGTGTTG | GGTTCTCCTTGGTGCTGCTGTAG |
| CPT1 | MW588015 | CGTGAGGAGGTTAAGCCGATGATG | GCCAGGTATCCGTGTCGTGTTG |
| CPT2 | MW588016 | GCAGCAGGGTGAGGAACGATTG | CACGATGAGGCTCAGGGATTTGTC |
| UCP1 | MW591774 | GCTTCGCTGTTCTTGGAATCTT | CCAGAGGGAAGGTGATCATGTC |
| UCP2 | MW591775 | GGATTGGCTGCCGCTTACTGG | CGCCTGGAATCGAACCTTCACC |

**Supplement Table 3. The sequences of primers used for real-time PCR analysis in 3T3-L1 cells.**

| Gene | Accession number | 5’ Primer Sequence | 3’ Primer Sequence |
| --- | --- | --- | --- |
| AdipoQ | NM_009605 | GCACTGGCAAGTTCTACTGCAA | GTAGGTGAAGAGAACGGCCTTGT |
| Cidea | NM_007702 | TGCTCTTCTGTATCG CCC AGT | GCCGTGTTAAGGAATCTGCTG |
| Cox5b | NM_009942 | GCTGCATCTGTGAAGAGGACAAC | CAGCTTGTAATGGGTTCCACAGT |
| Cox7a1 | NM_009944 | CAGCGTCATGGTCAGTCTGT | AGAAAACCGTGTGGCAGAGA |
| Cox8b | NM_007751 | GAACCATGAAGCCAACGACT | GCGAAGTTCACAGTGGTTCC |
| Dio2 | NM_010050 | CAGTGTGGTGCACGTCTCCAATC | TGAACCAAAGTTGACCACCAG |
| Fabp4 | XM_006530048 | ACACCGAGATTTCCTTCAAACTG | CCATCTAGGGTTATGATGCTCTTCA |
| Pgc1α | NM_008904 | CCCTGCCATTGTTAAGACC | TGCTGCTGTTCCTGTTTTC |
| Pparα | XM_011245517 | GCGTACGGCAATGGCTTTAT | GAACGGCTTCCTCAGGTTCTT |
| Pparγ | NM_001127330 | GTGCCAGTTTCGATCCGTAGA | GGCCAGCATCGTGTAGATGA |
| Prdm16 | XM_006539171 | CAGCACGGTGAAGCCATTC | GCGTGCATCCGCTTGTG |
| Ucp1 | NM_009463 | ACTGCCACACCTCCAGTCATT | CTTTGCCTCACTCAGGATTGG |
